# Knowledge and Attitudes toward First Aid among Medical and Nursing Students at Taibah University in Madinah City, Saudi Arabia: A Cross-Sectional Study

**DOI:** 10.3390/healthcare11222924

**Published:** 2023-11-08

**Authors:** Muayad Saud Albadrani, Abdulmajeed Mohammed Qashqari, Basel Abdulmonem Alqelaiti, Ohud Khalid Hammad, Raghad Khalid Hammad, Maram Salamah Alrehely, Walaa Abdulrahman Almeshhen, Emad Ali Albadawi

**Affiliations:** 1 Department of Family and Community Medicine, College of Medicine, Taibah University, Al-Madinah Al-Munawara 42353, Saudi Arabia; 2 College of Medicine, Taibah University, Al-Madinah Al-Munawara 42353, Saudi Arabiamaramalharbi1438@gmail.com (M.S.A.);; 3 Department of Anatomy, College of Medicine, Taibah University, Al-Madinah Al-Munawara 42353, Saudi Arabia

**Keywords:** first aid, medical education, nursing education, theoritical knowledge, practical knowledge, attitudes, Saudi Arabia

## Abstract

Objective: The objective of this study is to evaluate the first aid (FA) knowledge, practice, and attitude of medical and nursing students at Taibah University in Madinah. Methods: The study involved a cross-sectional online survey of 359 students from different academic years, using a revised and validated questionnaire on FA procedures, which were assessed utilizing a revised iteration of a questionnaire that had been previously validated. Results: Regarding the knowledge score outcomes, the median score was 4, with an interquartile range of (3,5). Approximately 32.3% of participants demonstrated an excellent level of knowledge in first aid. Age exhibited a substantial and positive correlation with knowledge scores (*p* < 0.001), no significant correlation was observed between age and practice scores (*p* = 0.782), whereas age exhibited a significant and positive relationship with attitude scores (*p* < 0.001). Switching to the practice score results, the median practice score was 3, with an interquartile range of 2 to 3. A considerable 39.6% of participants displayed a good level of practice, representing the highest percentage among students. In the context of attitude score findings, the median attitude score was 4, and the interquartile range was 3 to 4, this suggests that the majority of the participants had a positive attitude towards first aid and its importance. Around 27.6% of participants portrayed a good attitude level, followed by 27% who demonstrated an acceptable attitude level. In addition, gender emerged as a differentiating factor in the three primary outcomes, as females achieved superior results across all aspects. Conclusion: A significant proportion of medical and nursing students at Taibah University have solid FA knowledge, practice, and attitude. Age and education level reflect the impact of FA training and certification, which should be mandatory for all medical students. Further studies are needed to generalize the findings to other contexts.

## 1. Introduction

First aid (FA) refers to the initial medical assistance administered whether by a healthcare professional or ordinary person at the scene of an accident or to an individual who is injured or critically ill, before the arrival of an ambulance or additional medical help [1]. The objective of first aid is to safeguard life and hinder the deterioration of a patient’s condition until professional health care is accessible, in addition to the promotion of fast recovery for people who receive FA compared to people who do not. Any individual can initiate first aid actions during an emergency, requiring minimal or even almost no equipment [2]. The responsibility of healthcare units, such as paramedics and emergency medical technicians, is to deliver the necessary first aid required for the particular situation, to minimize patient distress, and to diminish the likelihood of disability and premature mortality [3,4]. Bystanders may also play an important role as they are supposed to call an emergency and perform basic actions with the available materials in the surroundings as sometimes the bystanders may help in saving the life of the injured person [5]. Moreover, FA is a vital skill that can have a positive impact on public health by preventing infections, reducing injuries, saving lives, improving chronic conditions, and empowering disadvantaged groups. FA teaches people how to protect themselves and others from infectious diseases, such as COVID-19, by following behaviors such as hand washing, mask-wearing, and physical distancing. FA also provides immediate and appropriate care for injuries and illnesses, such as bleeding, burns, fractures, and poisoning, before professional help arrives. In some cases, FA can restore breathing and circulation in someone who has stopped breathing or whose heart has stopped beating, using techniques such as chest compressions, rescue breaths, and automated external defibrillators (AEDs). Additionally, FA can help people who suffer from chronic conditions, such as diabetes, asthma, and epilepsy, to manage their symptoms and prevent complications. Finally, FA can reduce health inequalities by empowering people from low-income, rural, or marginalized communities to take charge of their health and well-being.

Despite the importance of knowledge regarding FA, it is noted that people worldwide lack the knowledge to help in saving lives whether they are medical or non-medical students. In a study made in Pakistan about FA in the management of burns, it was found that both medical and non-medical students lack knowledge about how to deal with such a situation [6]. It is usually the case to identify a degree of deficiency in the general public knowledge of FA [7]. However, future healthcare providers are expected to have higher levels of knowledge and practice in first aid, as they have a professional and ethical responsibility to provide effective and timely care to the society

Other important certifications for knowledge about FA are Basic Life Support (BLS) and Advanced Cardiovascular Life Support (ACLS). BLS focuses on providing immediate assistance to individuals facing life-threatening emergencies, such as cardiac arrest, choking, or drowning. It involves basic interventions aimed at maintaining the person’s airway, breathing, and circulation until advanced medical help arrives [8,9]. On the other hand, ACLS is more advanced healthcare for medical professionals, which is built upon BLS principles [10]. Both BLS and ACLS training are crucial components of the first aid continuum [11]. The difference between FA and BLS is that FA is a general term for the skills and knowledge needed to help someone who is injured or ill, while BLS is a specific type of FA that focuses on restoring breathing and circulation in someone who has stopped breathing or whose heart has stopped beating. FA can include things like bandaging wounds, treating burns, splinting fractures, and preventing shock. BLS can include things like performing chest compressions, giving rescue breaths, using an AED, and clearing an obstructed airway. FA is usually taught to the general public, while BLS is usually taught to healthcare professionals or people who work in high-risk environments.

FA training tends to be overlooked in medical education programs. Numerous studies have highlighted unsatisfactory outcomes concerning the preparedness of medical students and trainees in the field of FA [12]. Earlier investigations have indicated that healthcare practitioners possess restricted understanding and abilities when it comes to executing first aid procedures. Furthermore, certain studies have conveyed that healthcare professionals hold an unfavorable viewpoint about engaging in first aid due to a deficiency of self-assurance and proficiency in executing the required protocols [12,13]. Additionally, particular studies have unveiled disparities in the comprehension and viewpoints of healthcare practitioners, influenced by factors like gender, educational background, and prior exposure to first aid [14]. This study aims to evaluate and understand the knowledge, practice, and attitude of medical and nursing students toward first-aid measures. The results of this study will provide valuable information for medical and nursing schools to improve their curricula and for healthcare organizations to develop effective training programs for their staff.

## 2. Methods

This cross-sectional study was conducted at Taibah University for medical and nursing students in Madinah City, Saudi Arabia. The cross-sectional study was used with an online questionnaire with the data collection endpoint at the end of May 2023. A total of 359 students participated in the questionnaire out of a total of 1483 students who were studying medicine or nursing at Taibah University. We used the public service of creative research systems survey software [15], which was designed to create and conduct surveys, analyze data, and generate reports. We used this software to determine the precise population target of the sample size; it supported various types of surveys, such as web, email, phone, paper, and mobile surveys. It also offered a range of features, such as questionnaire design, sample management, data entry, data analysis, data visualization, and report writing. We found out that the proper sample size would be 305 participants at a 95% confidence interval, 5% margin of error, and 0.05 level of significance. This sample size was increased to 359. We administered a total of 500 questionnaires to the students of medicine and nursing at Taibah University. We used a stratified random sampling method to select the participants based on their college, educational level, and gender. We distributed the questionnaires online using Google Forms and sent reminders via email and WhatsApp to increase the response rate. Out of the 500 questionnaires that we administered, we received 359 valid responses, which gave us a response rate of 71.8%. We excluded responses that were incomplete, inconsistent, or duplicated. We considered this response rate to be satisfactory and representative of the target population.

An adapted edition of a questionnaire that was previously verified was employed to evaluate knowledge, practice, and attitude regarding first aid [16]. The final questionnaire was in English and consisted of 26 items, including 11 questions about demographic characteristics, 5 questions about theoretical knowledge with 1 right answer, 4 questions about practical knowledge with 1 right answer, and finally 5 questions about attitude, with the first 3 questions as descriptive questions and the last 2 questions with the answer yes or no, as shown in the Supplementary File S1.

### Statistical Methods

Data were analyzed using IBM SPSS (Statistical Package for Social Sciences), version 21. Numerical data were described as the median and interquartile range (IQR), while categorical data were described as numbers and percentages. Testing for normality was performed using the Kolmogorov–Smirnov test and the Shapiro–Wilk test. The median differences of total scores within each independent variable were tested using the Kruskal–Wallis Test. Spearman’s rank correlation test was used to determine the correlation between numeric variables and total scores. Furthermore, a 2-sided *p* value < 0.05 was considered statistically significant.

## 3. Results

In this section, we present the results of our cross-sectional survey on the knowledge, practice, and attitude of first aid among students of Taiba University. We used a questionnaire consisting of three sections: theoretical knowledge, practical skills, and attitude toward first aid. We scored each section based on the number of correct answers and categorized the levels of knowledge, practice, and attitude as poor, acceptable, good, or excellent. We then analyzed the data using descriptive and inferential statistics to examine the association between the scores and various demographic and academic variables, such as gender, age, college, educational level, monthly income, residence, marital status, first aid courses, first aid activities, BLS or ACLS certifications, and first aid experience. We also identified the most common errors and misconceptions in the theoretical knowledge and practical skills sections.

### 3.1. Participants’ Characteristics

The study enrolled 359 students from Taiba University, with a median age of 21 years and an interquartile range of 20 to 22. Among the participants, 59.3% were female, and 97.2% were single. The majority, comprising 89.4% of the total population, were enrolled in medical college. Among the different academic years, second-year students had the highest participation rate, while interns constituted only nine participants. Regarding the monthly income of the family, the largest proportion (66.6%) reported an income exceeding 10,000 SAR, with 11.4% reporting less than 5000 SAR, and 22% falling in the 5000 to 10,000 SAR range. In terms of residence, an overwhelming 99.4% of participants lived in the Madinah Region. Among all participants, 61% had received formal FA training, and 45.7% had engaged in volunteer work or participated in FA activities. Additionally, 44.3% held certifications in Basic Life Support (BLS) or Advanced Cardiovascular Life Support (ACLS), and 40.9% had encountered situations that necessitated the application of FA techniques, as shown in Table 1.

### 3.2. Results of Knowledge Score

**Figure 1:**
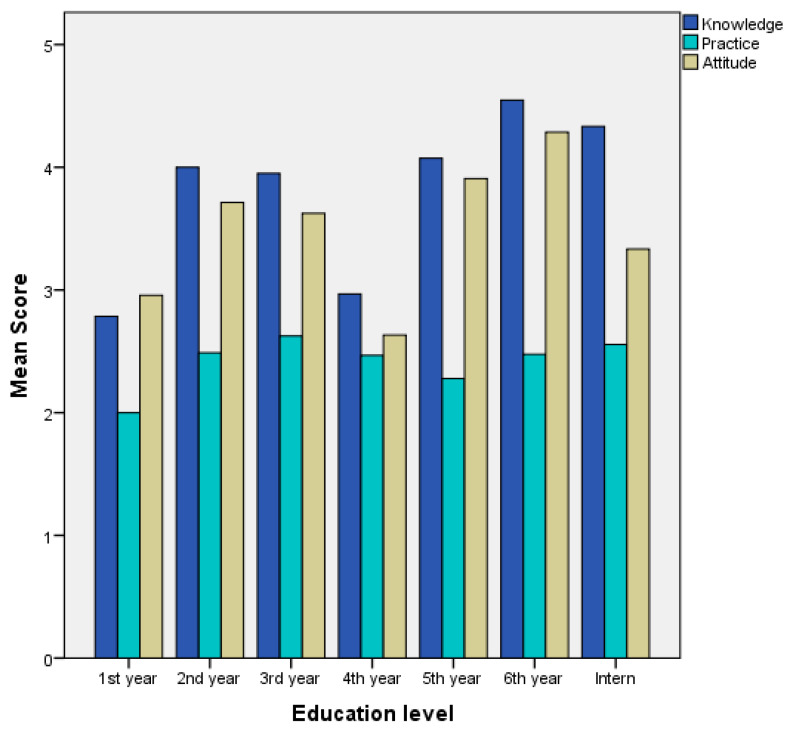
Comparing mean score of knowledge, practice, and attitude on first aid among students.

The median score for knowledge was four, with an interquartile range of (3,5). Around 32.3% of the participants exhibited an excellent level of knowledge of FA, while 31.8% demonstrated a good level. As shown in Table 2, a significant disparity was observed in the mean ranking of knowledge scores between genders, favoring females (*p* < 0.001). A statistically significant variance in knowledge scores existed among different educational levels (*p* < 0.001), as fifth-year and sixth-year students, and interns achieved higher scores compared to other grades (Figure 1). Additionally, a notable discrepancy in knowledge scores was evident between participants in Madinah and those residing in other regions, with the former having higher scores (*p* = 0.013). There were significant differences in knowledge scores between participants who had taken first aid courses and those who had not (*p* < 0.001), as well as between those who engaged in first aid activities and those who did not (*p* < 0.001). Likewise, a significant difference existed in knowledge scores between participants who encountered cases necessitating first aid and those who did not (*p* < 0.001). However, no statistically significant differences in knowledge scores were found concerning college, monthly income, marital status, or possession of BLS or ACLS certifications (*p* = 0.765, *p* = 0.942, *p* = 0.692, and *p* = 0.151, respectively). As shown in Table 3, the highest percentage of wrong answers in the theoretical knowledge section was related to the last question “Does first aid require expensive equipment?” as 43.7% answered this question in a wrong way. Supplementary Table S1 further shows that age demonstrated a significant positive correlation with knowledge scores (*p* < 0.001).

### 3.3. Results of Practice Score

The median score of practice score was 3 (IQR 2,3). A total of 39.6% of the participants had a good level of practice, which was the highest percentage of students. Table 2 shows that there was a significant median difference in the practice score between females and males, favoring the females (*p* = 0.001). There was a statistically significant difference in the mean rank of the practice score among the different categories of educational level and monthly income (*p* = 0.020, *p* = 0.048), respectively. However, there was no statistically significant difference in the practice score in terms of college, marital status, taking first aid courses, participating in first aid activities, having BLS or ACLS, and encountering a case that required first aid (*p* = 0.202, *p* = 0.579, *p* = 0.773, *p* = 0.553, *p* = 0.137, and *p* = 0.310, respectively). Table 4 shows that in the first question in the practice section “What would be your first step if you encountered a person with profuse leg bleeding due to a gunshot wound?”, the percentage of wrong answers was higher than right answers, with only 40.9% of correct answers. Supplementary Table S1 shows that there was no significant correlation between age and practice score (*p* = 0.782).

### 3.4. Results of Attitude Score

The median attitude score was 4 (IQR 3,4), and 27.6% of the participants had a good level of attitude, followed by 27% who had an acceptable level of attitude. Table 2 shows that there was a significant median difference in the attitude score between females and males, favoring females (*p* < 0.001). There was a statistically significant difference in the mean rank of the attitude score among the different categories of educational level and current living city, between participants who took first aid courses and those who did not, between participants who encountered a case that required first aid and those who did not, and between participants who participated in first aid activity and those who did not (*p* < 0.001, *p* = 0.013, *p* = 0.008, *p* < 0.001, and *p* < 0.001), respectively. On the other hand, there was no statistically significant median difference in the attitude score in terms of college, monthly income, and having BLS or ACLS (*p* = 0.344, *p* = 0.228, *p* = 0.755, *p* = 0.081, respectively). Table 5 shows that in the third and fourth questions in the attitude section, “Would you like to give a basic idea of first aid techniques to your fellow students?” and “Do you think first aid decreases the burden of hospitals?”, only half the participants provided correct answers. Supplementary Table S1 shows that there was a significant positive correlation between age and attitude score (*p* < 0.001).

## 4. Discussion

In this study, we conducted a cross-sectional survey to evaluate and understand the knowledge, practice, and attitude of medical and nursing students toward first-aid measures. We found that both groups had a moderate level of knowledge, practice, and attitude toward first aid, but there were some significant differences between them. Medical students had higher knowledge and practice scores than nursing students, while nursing students had higher attitude scores than medical students.

These findings suggest that medical students have more theoretical and practical exposure to first aid than nursing students, but nursing students have more positive and supportive views on first aid than medical students. We also found that female participants, older participants, and participants who had taken first aid courses, participated in first aid activities, or encountered first aid cases had higher scores in all aspects of first aid than their counterparts.

We concluded that the medical and nursing students of Taiba University in Madinah have good potential to perform first aid, but they need more education and training to improve their skills and confidence. We also recommend that first aid should be integrated into the curriculum of both medical and nursing colleges and that more opportunities for first aid practice and simulation should be provided for the students.

Medical field students have higher knowledge about FA than the public because they study subjects related to the human body and its functions, they learn and practice FA skills in clinical scenarios and cases, and they are motivated and interested in FA as part of their professional development and responsibility. However, our study showed that their knowledge is not sufficient, and they need more education and training in FA. Therefore, it is advisable to integrate first-aid training programs into the curricula of medical schools and colleges. This integration would empower medical students to contribute more effectively to the well-being of society [17,18].

We also found that 61% of participants, which is the majority, took FA courses previously, which is higher than other cross-sectional studies that were evaluated in Suadi Arabia in another university [19]. Additionally, 44.3% had previous BLS or ACLS, which is relatively higher than what Alanazi et al. revealed in their study [12]. In the study conducted by Batais et al. [20], it was found that 61.8% of their Saudi Arabian sample encountered situations necessitating first aid intervention. Similarly, our study revealed that 40.9% of participants faced cases demanding first aid applications. This underscores the significance of ensuring that these participants possess adequate knowledge to effectively mitigate injuries and reduce morbidity. Abbas et al. [21] confirmed that trained medical students’ answers to the questionnaire were better than the untrained students, which is similar to our results as our study showed a significant difference in knowledge and attitude for the students who participated in activities that required FA or had already taken FA courses. On the other, Abbas et al. mentioned that less than half the population was found to have previous training, which was conducted in 2011; we found a higher percentage in our medical student population with previous training on FA (61%), which may represent an increase in the medical students with previous training. We also noticed a significant difference in the percentage of medical students who took previous FA, especially compared with the general population, as Bashekah et al. [22] reported that only 36% had previous training related to FA. Bashekah et al. also reported that even a third of the participants who took previous training for FA did not have sufficient knowledge, which shows the necessity of ongoing training.

One of our limitations is that we did not set any time limits for completing the questionnaire, as we wanted to give the students enough time and flexibility to answer the questions at their own pace and convenience. However, this also posed a limitation for our study, as it may have facilitated answering the questions with the help of the internet. This means that some students may have searched for the answers online or consulted other sources or people while completing the questionnaire. This could have affected the validity and reliability of the data, as it may not reflect the true knowledge, practice, and attitude of the students towards first aid. It could also introduce a bias or error in the data, as some students may have answered more accurately or confidently than others who did not use any external help. We acknowledge this limitation and suggest that future research should use more secure and controlled methods to administer the questionnaire, such as setting time limits, using passwords, or using proctoring software. This would prevent or reduce the possibility of answering the questions with the help of the internet and ensure that the data are more valid and reliable. The outcomes of this study should not be extrapolated to encompass all medical colleges or different regions within Saudi Arabia as there may be differences in the characteristics, backgrounds, and experiences of the students from different universities or regions. Also, our study used a cross-sectional design, which means that we collected data from the participants at one point in time. Therefore, our results may not reflect the changes or trends in the knowledge, practice, and attitude of first aid among the students over time, as there may be factors that influence their learning and performance of first aid, such as new guidelines, policies, technologies, or events. For example, some students may have improved or worsened their knowledge, practice, and attitude toward first aid after completing our questionnaire due to their exposure to new information, training, or situations. Moreover, most of our included participants were medical students and there were fewer nursing students. This is because most of the data collectors were from the medical college. We hope that this study will inspire educational authorities to contemplate measures such as implementing compulsory foundational first aid courses to help medical students have a higher effect on society.

## 5. Conclusions

Our cross-sectional study revealed that a significant portion of medical and nursing students at Taibah University in Madinah exhibit good to excellent knowledge, practice, and attitude in FA. Female participants outperformed their male counterparts, and there is a positive correlation between age and knowledge, emphasizing the educational impact. Comparisons with previous research demonstrate a higher prevalence of FA training and certification among our participants. This underscores the growing recognition of the importance of first aid education. However, our findings should be applied with caution, as they pertain specifically to our study population.

To strengthen preparedness and response, it is recommended that medical and nursing education institutions consider introducing mandatory FA courses. Further studies are essential to gain a more comprehensive understanding of FA readiness among medical and nursing students, and the general population.

## Figures and Tables

**Table 1:** Baseline characteristics of the participating students.

Characteristics	N	Percentage
**Gender**		
Female	59.3	213
Male	40.7	146
**Age**		
Median (IQR)		21 (20.2)
**College**		
Medical College	321	89.4
Nursing College	38	10.6
**Educational level**		
1st Year (Preparatory Year)	70	19.5
2nd Year	84	23.4
3rd Year	40	11.1
4th Year	60	16.7
5th Year	54	15
6th Year	42	11.7
Intern	9	2.5
**Monthly income**		
<5000 SAR	41	11.4
5000–9999 SAR	79	22
10,000–20,000 SAR	127	35.4
>20,000 SAR	112	31.2
**Current Living**		
Madinah Region	357	99.4
Others	2	0.6
**Marital status**		
Single	349	97.2
Married	9	2.5
widowed	1	0.3
**Have you taken first-aid courses**		
Yes	219	61.0
No	140	39.0
**Have you volunteered/participated in activities that require first aid**		
Yes	164	45.7
No	195	54.3
**Did you have BLS or ACLS?**		
Yes	159	44.3
No	200	55.7
**Have you encountered a case that requires first aid?**		
Yes	147	40.9
No	212	59.1

**Table 2:** Distribution of levels of knowledge, practices, and attitude on first aid among students.

	N (%)
**Knowledge score: Median (IQR)**	**4 (3,5)**
**Level of knowledge**	
Poor	67 (18.6)
Acceptable	62 (17.3)
Good	114 (31.8)
Excellent	116 (32.3)
**Practice score: Median (IQR)**	**3 (2,3)**
**Level of practices**	
Poor	74 (20.6)
Acceptable	103 (28.7)
Good	142 (39.6)
Excellent	40 (11.1)
**Attitude score: Median (IQR)**	**4 (3,4)**
**Level of attitude**	
Poor	75 (20.9)
Acceptable	97 (27)
Good	99 (27.6)
Excellent	88 (24.5)

**Table 3:** Statistical median and mean rank differences of knowledge in relation to the sociodemographic characteristics.

Characteristics	Knowledge Score (5)		*p* Value *
	Median (IQR)	Mean Rank	
**Gender**			
Female	4 (3,5)	204	<0.001
Male	4 (2,4)	143	
**College**			
Medical College	4 (3,5)	179	0.765
Nursing College	4 (3,5)	184	
**Edu** **cation level**			
1st Year (Preparatory Year)	3 (2,4)	110	<0.001
2nd Year	4 (3,5)	200	
3rd Year	4 (3,5)	197	
4th Year	3 (2,4)	135	
5th Year	4 (4,5)	210	
6th Year	5 (4,5)	254	
Intern	4 (4,5)	227	
**Monthly income**			
<5000 SAR	4 (3,5)	186	0.492
5000–10,000 SAR	4 (3,4)	164	
10,000–20,000 SAR	4 (3,5)	184	
>20,000 SAR	4 (3,5)	183	
**Current Living**			
Madinah Region	4 (3,5)	180	0.013
Others	0	4	
**Marital status**			
Single	4 (3,5)	180	0.692
Married	4 (2,5)	171	
widowed	3	98	
**Have you taken first-aid courses**			
Yes	4 (4,5)	206	<0.001
No	3 (2,4)	139	
**Have you volunteered/participated in activities that require first aid**			
Yes	4 (4,5)	207	<0.001
No	4 (2,5)	157	
**Did you have BLS or ACLS?**			
Yes	4 (3,5)	188	0.151
No	4 (3,5)	173	
**Have you encountered a case that requires first aid?**			
Yes	4 (4,5)	218	<0.001
No	4 (2,4)	153	

* Kruskal–Wallis Test.

**Table 4:** Statistical median and mean rank differences of practices in relation to the sociodemographic characteristics.

Characteristics	Practice Score (4)		*p* Value *
	Median (IQR)	Mean Rank	
**Gender**			
Female	3 (2,3)	195	0.001
Male	2 (1,3)	158	
**College**			
Medical College	3 (2,3)	182	0.202
Nursing College	2 (1,3)	160	
**Edu** **cation level**			
1st Year (Preparatory Year)	2 (1,3)	144	0.020
2nd Year	3 (2,3)	188	
3rd Year	3 (2,3)	208	
4th Year	3 (2,3)	187	
5th Year	2 (2,3)	170	
6th Year	3 (2,3)	192	
Intern	3 (2,3)	198	
**Monthly income**			
<5000 SAR	2 (1,3)	164	0.048
5000–10,000 SAR	2 (1,3)	161	
10,000–20,000 SAR	3 (2,3)	197	
>20,000 SAR	2 (2,3)	178	
**Current Living**			
Madinah Region	3 (2,3)	180	0.013
Others	0	7	
**Marital status**			
Single	3 (2,3)	179	0.579
Married	3 (2,3)	209	
widowed	2	126	
**Have you taken first-aid courses**			
Yes	3 (2,3)	181	0.773
No	2 (2,3)	178	
**Have you volunteered/participated in activities that require first aid**			
Yes	3 (2,3)	183	0.553
No	2 (2,3)	177	
**Did you have BLS or ACLS?**			
Yes	2 (2,3)	171	0.137
No	3 (2,3)	186	
**Have you encountered a case that requires first aid?**			
Yes	3 (2,3)	186	0.310
No	2 (2,3)	175	

* Kruskal–Wallis Test.

**Table 5:** Statistical median and mean rank differences of attitude in relation to the sociodemographic characteristics.

Characteristics	Attitude Score (5)		*p* Value *
	Median (IQR)	Mean Rank	
**Gender**			
Female	4 (3,5)	203	<0.001
Male	3 (2,4)	145	
**College**			
Medical College	4 (3,4)	178	0.344
Nursing College	4 (3,5)	194	
**Edu** **cation level**			
1st Year (Preparatory Year)	3 (2,4)	138	<0.001
2nd Year	4 (3,4)	196	
3rd Year	3.5 (3,5)	189	
4th Year	2 (1,4)	119	
5th Year	4 (3,5)	214	
6th Year	5 (4,5)	250	
Intern	3 (2,5)	164	
**Monthly income**			
<5000 SAR	4 (3,5)	203	0.228
5000–10,000 SAR	3 (3,4)	170	
10,000–20,000 SAR	3 (2,4)	172	
>20,000 SAR	4 (3,5)	187	
**Current Living**			
Madinah Region	4 (3,4)	180	0.013
Others	0	3	
**Marital status**			
Single	4 (3,4)	179	0.755
Married	4 (2,5)	196	
widowed	3	124	
**Have you taken first-aid courses**			
Yes	4 (3,5)	191	0.008
No	3 (3,4)	162	
**Have you volunteered/participated in activities that require first aid**			
Yes	4 (3,5)	203	<0.001
No	3 (3,4)	160	
**Did you have BLS or ACLS?**			
Yes	4 (3,5)	190	0.081
No	3 (3,4)	171	
**Have you encountered a case that requires first aid?**			
Yes	4 (3,5)	207	<0.001
No	3 (3,4)	160	

* Kruskal–Wallis Test.

## Data Availability

Data are available upon request.

## Supplementary Materials

The following supporting information can be downloaded at: https://www.mdpi.com/article/10.3390/healthcare11222924/s1, Table S1: Frequency and percentage of knowledge, practices, and attitude on first aid among Students. File S1: Questions about First Aid knowledge, attitude, and practice in the questionnaire.
