# Supplementary material for: Knowledge and Attitudes toward First Aid among Medical and Nursing Students at Taibah University in Madinah City, Saudi Arabia: A Cross-Sectional Study
Source: Healthcare (Basel). 2023 Nov 8;11(22):2924. doi: 10.3390/healthcare11222924 (PMC10671620; doi:10.3390/healthcare11222924)
Supplement: Supplementary file 1 [file healthcare-11-02924-s001.zip › healthcare-2640689-supplementary.docx]

**Supplementary Table 1: Frequency and percentage of knowledge, practices, and attitude on first aid among Students.**

| **False**  **N (%)** | **True**  **N (%)** | **Theoretical Knowledge** |
| --- | --- | --- |
| 85 (23.7) | 274 (76.3) | 1. What does CPR stand for? |
| 108 (30.1) | 251 (69.9) | 2. Which of the following is the correct ratio of chest compression? |
| 52 (14.5) | 307 (85.5) | 3. What is the proper definition of an open fracture? |
| 76 (21.2) | 283 (78.8) | 4. Why should you act quickly for a casualty with severe bleeding? |
| 157 (43.7) | 202 (56.3) | 5. Does first aid require expensive equipment? |
| **False**  **N (%)** | **True**  **N (%)** | **Practice statement** |
| 212 (59.1) | 147 (40.9) | 1. What would be your first step if you encountered a person with profuse leg bleeding due to a gunshot wound? |
| 125 (34.8) | 234 (65.2) | 2. What would you do first for a person who has burnt his hand? |
| 148 (41.2) | 211 (58.8) | 3. What would you encourage your colleague to do if he were to become distressed by a piece of food lodged in his airway? |
| 99 (27.6) | 260 (72.4) | 4. What should you do to a person who has fallen down with a suspected thigh fracture? |
| **False**  **N (%)** | **True**  **N (%)** | **Attitude statement** |
| 109 (30.4) | 250 (69.6) | 1. If you have good knowledge of first aid, you should not hesitate to use it when needed? |
| 25 (7.0) | 334 (93.0) | 2. Do you support including first aid in the medical college curriculum? |
| 184 (51.3) | 175 (48.7) | 3. Would you like to give a basic idea of first aid techniques to your fellow students? |
| 169 (47.1) | 190 (52.9) | 4. Do you think first aid decreases the burden of hospitals? |
| 65 (18.1) | 294 (81.9) | 5. Do you think first aid increases patients' survival rates? |
