# Supplementary material for: Knowledge and Attitudes toward First Aid among Medical and Nursing Students at Taibah University in Madinah City, Saudi Arabia: A Cross-Sectional Study
Source: Healthcare (Basel). 2023 Nov 8;11(22):2924. doi: 10.3390/healthcare11222924 (PMC10671620; doi:10.3390/healthcare11222924)
Supplement: Supplementary file 1 [file healthcare-11-02924-s001.zip › healthcare-2640689-supplementary.pdf]

Supplementary Table 1: Frequency and percentage of knowledge, practices, and attitude on first aid among Students.

Theoretical Knowledge	True N (%)	False N (%)
1. What does CPR stand for?	274 (76.3)	85 (23.7)
2. Which of the following is the correct ratio of chest compression?	251 (69.9)	108 (30.1)
3. What is the proper definition of an open fracture?	307 (85.5)	52 (14.5)
4. Why should you act quickly for a casualty with severe bleeding?	283 (78.8)	76 (21.2)
5. Does first aid require expensive equipment?	202 (56.3)	157 (43.7)
**Practice statement**	**True N (%)**	**False N (%)**
1. What would be your first step if you encountered a person with profuse leg bleeding due to a gunshot wound?	147 (40.9)	212 (59.1)
2. What would you do first for a person who has burnt his hand?	234 (65.2)	125 (34.8)
3. What would you encourage your colleague to do if he were to become distressed by a piece of food lodged in his airway?	211 (58.8)	148 (41.2)
4. What should you do to a person who has fallen down with a suspected thigh fracture?	260 (72.4)	99 (27.6)
**Attitude statement**	**True N (%)**	**False N (%)**
1. If you have good knowledge of first aid, you should not hesitate to use it when needed?	250 (69.6)	109 (30.4)
2. Do you support including first aid in the medical college curriculum?	334 (93.0)	25 (7.0)
3. Would you like to give a basic idea of first aid techniques to your fellow students?	175 (48.7)	184 (51.3)
4. Do you think first aid decreases the burden of hospitals?	190 (52.9)	169 (47.1)
5. Do you think first aid increases patients' survival rates?	294 (81.9)	65 (18.1)
